# Challenges of modern work environments and means of overcoming them in the context of psychosocial risk assessments

**DOI:** 10.1186/s12889-024-20818-w

**Published:** 2024-12-06

**Authors:** Valeria Pavlista, Peter Angerer, Mathias Diebig

**Affiliations:** 1 Institute for Occupational, Social and Environmental Medicine, Centre for Health and Society, Medical Faculty, Heinrich-Heine University, Moorenstr. 5, 40225 Düsseldorf, Germany; 2 Department of Work and Organizational Psychology, Faculty I - Psychology, Trier University, Universitätsring 15, 54296 Trier, Germany

**Keywords:** Modern work environments, New work, Psychosocial risk assessment, Mental health, Employee wellbeing, Occupational measures

## Abstract

**Background:**

Modern work environments constitute an underrepresented part of psychosocial risk assessments at work. Little is known about whether there is an increased stress load at all and what possible improvements could be made in such a case.

**Methods:**

Modern work environments were assessed in an online questionnaire in 33 companies across a period of more than 4 years. A total of 3431 employees participated in the study. Both quantitative and qualitative data was applied to obtain a differentiated picture.

**Results:**

Increased stress caused by modern work environments was an issue for around a third of the sample. 31.6% of the participants at least sometimes struggled to balance work and private life. Quite a few of the participants (36.3%) worked sometimes or more outside regular working hours. For 32.4% of participants, the workload has increased due to new technologies, but for 30.4% it has not. The majority (81.4%) feel they can work productively in home offices. The data from 178 completed free text fields on improving modern work environments from the employees’ perspective was analysed. Many named suggestions relate to improvements in time management.

**Conclusion:**

This study provides both detailed insights into various aspects of modern work environments and offers solutions to counteract possible negative consequences. Assessing modern work environments in psychosocial risk assessments would be a valuable addition to its completeness.

## Background

The world of work is in a state of constant flux, with profound changes unfolding in response to technological advancements. While these changes have brought forth unprecedented opportunities and efficiencies, they have also given rise to a unique set of challenges that significantly impact the well-being of individuals in the workforce [1]. Continuous accessibility to work-related communications, such as emails and messages has led to an unceasing connectivity, creating challenges like disengaging from work and emotional exhaustion. As the traditional boundaries of the workplace become more fluid, the concept of work and life balance undergoes a transformation. Remote work, facilitated by technological innovations, has enabled greater flexibility but also blurred the lines between personal and professional life [2]. This development has become a major topic for occupational health research in order to understand the connections and consequences for the health of the working population. There is no doubt that professional and private life influence each other, but the direction and, above all, the effects of negative health consequences are different and depend on the respective context [3–5]. It is known without doubt that a poor work-life balance can be harmful to workers’ health [6], but at the same time the flexibility of the modern working environment also offers new coping strategies [7]. Research has shown that new technologies can be both beneficial and burdensome to workers’ health [8, 9]. Moreover, there is not only little empirical knowledge about the consequences of modern work environments, but also little knowledge about how to deal with the effects of modern working environments in the context of organisational measures. Even though, for example, the negative consequences of work life balance (WLB) are well researched, there is a surprisingly little amount of research how to improve WLB with organisational measures [10]. Furthermore, not much information is available on what employees make of these interventions.

In order to adequately capture the effects of the new working environment, it is important that organizations have implemented suitable screening procedures so that any increased stress situations can be recognized in good time. However, the newest developments are often underrepresented in standard screening tools. For example, although screening for psychosocial stress is very significant, it is not commonly assessed in organizational practice [11]. Since dealing with a highly complex and ambiguous situation, it is necessary to look closely at the causes and the context of the situation to understand the big picture. As psychosocial risk assessment (PRA) is the most frequently used tool for the systematic assessment of working conditions in the workplace, it would be of the greatest benefit to workers’ health if it were regularly updated with the newest occurring issues.

It has been demonstrated that PRA and other organisational interventions work best, if employees are included in the process [12–15]. This involves not only analysing the general stress levels of employees in the first step of PRA, but also asking them what they think would help to improve the situation, and involving them in the decision-making, design and implementation of the measures taken [12]. As the employees are typically affected by the measures and they know the challenges of their work environment well, they should be involved especially in the measure selection, development, and implementation [16]. This would not only improve compliance, but also lead to a better understanding of what could help. The positive effects of employee participation range from a higher probability of intervention-success, increased motivation, employee job satisfaction, employee commitment and productivity [17–20].

The purpose of this study is to find out whether modern work environments are a relevant issue for worker’ health and therefore if it should be addressed in screenings and organisational interventions. Furthermore, this study strengthens the employees’ perspective and involves them by asking them what they regard as critical and supportive. By analysing these aspects, this research endeavours to shed light on unrepresented topics of research that are nevertheless important to people’s health. This study is aiming to support policymakers, employers, and healthcare professionals in their pursuit of creating work environments that foster the psychological and emotional welfare of the workforce. The methodological approach of this work combines both quantitative and qualitative methods to get a differentiated, in-depth view of the situation. The quantitative parts provide descriptive statistics on key trends in the sample that allow conclusions about the distribution in the general population. The qualitative part allows for a deeper exploration of the employees’ perspective and new approaches to stress induced by modern work environments. Employee participation is taken into account by giving them a voice and analysing their views on the causes of stress and solutions to avoid it.

### Modern work environments

The current picture of studies investigating mental health in the context of modern work environments reveal a complex overall situation. Unarguably, the digitalization has an impact on mental health with many different factors to be considered [21, 22]. With the spread of digitalization, the work environment is evolving, new stressors are emerging and the perception and appraisal of stress is changing [23]. The term modern work environments was deliberately chosen, because it covers a wide range of aspects and, in the context of PRA, must include the company’s situation and what it considers to be “new” and “modern”. This means that the technology itself does not have to be generally new but was relatively recently introduced into company practice. For example, certain digital communication channels have been around for some time but may only have been recently implemented within a department. The term covers both the technical work equipment, and the social aspects induced by modern work environments. This study analyses four different dimensions of modern work environments that all contribute to the big picture: work life balance (WLB), new technologies, boundary permeability, and working from home (WFH).

### Work life balance

WLB is one aspect that has received much attention in the context of modern work environments over the years as it captures the aim of living in balance with personal and work life [24, 25]. It encompasses a person’s ability to manage their time, energy, and resources effectively, ensuring that they are neither overwhelmed by their work nor neglect their personal life. Achieving this equilibrium is vital for maintaining physical and mental health, fostering positive relationships, and nurturing personal growth. It has been demonstrated that an imbalance or conflict between work and personal aspects of life can negatively influence well-being and cause stress and burnout [4, 26–28]. It impacts workers’ job satisfaction and as a consequence, their job performance [29].

### New technologies

Increasing digitalization and emerging technologies, such as artificial intelligence and automation, transform industries by streamlining processes and increasing efficiency and therefore pose another main aspect of modern work environments. This has the potential to make work easier for employees by (partially) automating work steps, thus saving time, and simplifying communication channels. However, automation can come with job insecurity and the fear of a deterioration in the economic situation of individuals [30]. Digitalized communication channels are efficient in terms of time, they enable rapid exchange and lead to constant availability. This increases the pressure to respond quickly – a phenomenon also known as workplace telepressure [31]. As a result, employees must adapt to new forms of work and new forms of communication, which is a struggle for a part of the workforce [32]. As society becomes increasingly reliant on innovative tools and processes, it is essential to examine the influence of new technologies on working conditions and mental health. The continuous availability of information through digital platforms and the increasing demand for constant connectivity can lead to information overload [33]. Technology overload and invasion are factors that result from intensive technology use and have been shown to have a negative impact on workers’ health [33, 34].

### Boundary permeability

Another aspect of modern work environments is the blurring of the boundaries between personal life and work [35–37]. As technologies such as smartphones and laptops enable constant connectivity, it becomes increasingly difficult to maintain the boundaries between work and personal life. The traditional distinction between time and space reserved for both is no longer clearly possible. The automatic documentation of most communication tools not only shows when messages were sent, but also in real time whether and when users are online. Most employees are aware of the fact that supervisors or customers use tools that offer transparency but also the possibility to track them [38].

### Working from home

Moreover, with many companies offering flexible working arrangements, employees have the opportunity to work from home or even anywhere at any time. This is often in the interest of employees, as it gives them more flexibility, and allows them to take more responsibility for their time management. However, it usually means that employees no longer fall within the scope of their company’s occupational health and safety program and therefore bear the responsibility alone. For instance, most companies do not check the compliance with ergonomic principles when organising WFH equipment or the adherence of recovery times.

As with other modern work environment aspects, it is undecided whether WFH is problematic for the employees. Research shows that employees working remotely feel socially isolated and need more organisational support, though on the other hand are less emotionally exhausted and more satisfied with their job [39]. Although remote work offers more flexible time-management options, it may also lead to work intensification and the promotion of old role models (e.g. that women are mainly responsible for care work and men for earning an income), as it has been observed during the COVID-pandemic [40, 41]. WFH is beneficial to the workforce if workers have well-equipped, noise and distraction free workspaces. The positive aspects of such an working environment can be observed, for example, in the reduction of psychological and physical stress responses [42].

### Psychosocial risk assessments

The fundamental components of Psychosocial Risk Assessments (PRA) are outlined in the European framework for psychosocial risk management [43, 44]. In Germany, the Occupational Safety and Health Act obliges employers to design the job in a manner that minimizes the potential threat to life, as well as physical and mental well-being, and strive to reduce any residual risk to the lowest extent possible (Sect. 4 of the Occupational Safety and Health Act). The PRA process is described in detail in the Joint German Occupational Safety and Health Strategy [45]. The procedure follows a typical pattern for systematically assessing various aspects of working conditions at the occupational level [14]. The first steps of PRA are the definition of the area to be analysed with similar tasks and the inclusion of all relevant persons. Once a suitable measurement method has been found, the psychosocial risk is assessed. If problematic areas are discovered, countermeasures are developed and implemented. The effectiveness of those measures is evaluated, and the process is updated if necessary [14, 46]. The analysis methods in PRA are most commonly employee surveys but can alternate to observational interviews or facilitated workshops. Psychosocial risks addressed in PRA encompass factors such as work content, work organisation of work, working time, social relations, work equipment, and working environment [14]. Modern work environments are only implicitly included as they influence many of the named aspects. However, not many standard questionnaires include specific questions regarding modern work environments. For example, if employees experience high work intensity, one reason behind this could be that new technologies increase the speed of work by allowing new requests to reach employees quickly. By including specific questions relating to modern working environments, it could be easier and quicker to find the cause and therefore the solution to the high work intensity. We argue that modern work environments may cause psychosocial stress and therefore should be considered as one aspect of PRA.

### Measures to improve the impact of modern work environments

It has been demonstrated that organisational interventions can reduce occupational stress and its symptoms [47, 48]. Moreover, managing job demands can improve work engagement and mental health for example by reducing the risk of burnout [49, 50]. Brough et al. [10] reviewed organisational interventions to improve WLB. They identified several measures to help workers improve WLB: compressed workweeks, giving workers control over their work schedules, and choosing how shift work is scheduled. The involvement of employees in the organization of work has shown promising results [15, 51]. The recent COVID-pandemic has highlighted the importance of organizational measures that take into account modern work environments, such as remote work. To address the new situation, organizational measures must also be available to employees remotely. Digital tools offer such possibilities and thus represent a possible intervention method for modern work environments [52]. To date, there is little evidence of the effectiveness of health and safety measures that address the impact of modern work. Little is also known about whether these interventions are at all wanted by the employees. It can be assumed that organisational interventions would be very advantageous, as it has been shown that for example companies that offer WLB support are less likely to lose employees due to job satisfaction and work pressure [53]. Although the effectiveness has not been evaluated very often, there are a number of suggestions as to how such measures should be designed [54]. Generally, it is advised to track, record and limit working time. The work tasks should contain varied aspects such as planning, execution, and monitoring as well as allowing enough time and control to the employee to execute it [14]. It is advised to schedule breaks, respect recovery times, and guarantee an undisturbed rest period [55]. There are quite a few expert suggestions, how measures should be designed generally, but little suggestions from the employees’ perspective. We are hoping to close this gap with our research. Therefore, the employees’ perspective was chosen to find suitable organisational interventions addressing modern work environments, firstly because their participation should be enabled and, secondly because this perspective is underrepresented in research.

### Aims of the study

Several objectives are pursued with this manuscript. We want to….

1. Demonstrates that modern work environments can cause psychosocial stress and therefore should be included as an aspect of PRA.
2. Describe the severity of the stress load caused by modern work environments on a quantitative level.
3. Investigate the reasons for increased stress levels with both quantitative and qualitative methods
4. Explore employees’ suggestions for measures that target modern work environments

## Methods

### Study design and procedure

An online tool as well as an online questionnaire for PRA was developed in the Dynamik 4.0 project [56–58]. The questionnaire was developed specifically for the needs of the modern working world, especially with regard to digitalization in the new industry [58, 59]. The validation of the questionnaire showed good values with regard to reliability and validity [58].

The project provided companies with training and guidance by experts and the access to the tool to execute PRA. In consultation with PRA experts from the Dynamik 4.0 team, all companies first identified a department or an area with similar activities to be evaluated, and in the following designed a questionnaire to address the specific setting. The available questionnaire pool contained all aspects to be considered in PRA, but with the additional parts of dealing with modern working environments. All questions were structured according to the same pattern. First, the Screening questions were presented, e.g. it was assessed if a certain construct or stress factor was present (*“I find it difficult to balance my professional and private life”)*. All items were measured with a five-point Likert scale. In addition, participants could select “no answer” if they did not want to or could not give a meaningful answer [58]. Second, the Problem question was presented, if the stress factor was rated at least neutral or higher (if it was lower, the questionnaire continued with the next screening question). There were multiple select options to explain the reason behind it (“Option 1: “*Work commitments prevent me from spending time with my family/friends”*, Option 2: *“Private commitments keep me from work”*, etc. Please note that all possible options are listed in the corresponding results section). Moreover, there was also a free text field to explain the situation in the participants’ own words *(“Why do you find it difficult to balance your professional and private life?”*). Third, after each section the questionnaire continued with the Solution question, e.g. participants were asked for suggestions how to improve the situation *(“We asked you about work-life balance in the last section. What do you think could be improved in these areas?”).*

The university’s institutional ethic committee approved the procedure (reference No: 5562). All steps of the study including data collection, analyses, and interpretation contributing to this work comply with the ethical standards of the relevant national and institutional committees on human experimentation and with the Helsinki Declaration of 1975.

### Data collection

The data was collected by using the Online Platform Dynamik 4.0. The participation was voluntary and free of charge; all participants submitted written informed consent. The questionnaires were hosted on Limesurvey and stored anonymously. The platform was designed responsively to adapt to all screen sizes and thus accessible across all devices with internet. The surveys were conducted between February 2018 and May 2022. Recruitment for the implementation of PRA was carried out in various ways: personal networks of the project staff, articles in specialist media as well as recruitment via associations and cooperation with an external consultancy firm that also offered occupational health services in companies. Since each company designed its own questionnaire, the deployment of the modern work environment questions varied slightly. Although full PRA were carried out, exclusively the four modern work environment items were analysed for the purpose of this study (see more details in Table 2).

### Participants and company characteristics

A total of 33 companies participated in the study. Companies of all sizes were included in this sample, most of which were very large companies; see Table 1 for more details.

**Table 1:** Sample characteristics by sample size

Company size	Number of enterprises	Number of employees on average
Small	5	31.6
Medium	8	122.0
Large	8	467.1
Very large	12	5848.3
**Total**	**33**	**2274.3**

Notes: Company size categories by number of employees were: 10–49 small enterprise, 50–249 medium size enterprise, 250–999 large enterprise, > 1000 very large enterprise

The data of 3431 workers employed by one of the 33 companies was analysed. The participants came from a wide range of sectors, with the information and communication (21%), financial (20%), and manufacturing (18%) sectors most heavily represented. As the demographic data was voluntarily given at the end of the questionnaire, demographic data of all participants is not available (2629 participants volunteered to share personal information). For the highest transparency, we therefore report *N* for each value. On average, participants were 44.5 years old (*SD* = 10.6, modal value = 50, *N* = 2350), the youngest was 17, and the oldest 70. The participants were employed for 12.7 years on average in the present company (*SD* = 10.1, modal value = 1, *N* = 2356). Of the 2469 participants who revealed their gender, 56.1% were male and 43.9% were female. 34.6% of the participants had a university degree (*N* = 1186; bachelor, master, diploma, etc.), and 26.2% an apprenticeship (*N* = 900). A very small amount had no higher education (0,8%, *N* = 27), and a few had a doctorate degree (4%, *N* = 137). One fifth of the participants (19.1%, *N* = 458) had management responsibility. Nearly a quarter of the participants (21.6%, *N* = 504) regularly worked at the weekend.

### Analysis

In the first step, the quantitative results of the Screening questions from the survey were analysed. This provides information on how high the stress was and whether an increased strain situation was present. In the next step, the Problem questions including the additional free text fields were analysed, in order to better understand contributing factors of stress. Finally, the Solution questions to find out how to improve the situation from the employees’ point of view were analysed. We used IBM SPSS Statistics 28.0, Microsoft 365 Excel, and MAXQDA 2018 to perform the data analysis. We applied a mixed methods procedure with both quantitative and qualitative analyses to get as good an insight as possible into the underlying aspects of modern work environments. Content analysis was applied to structure and categorize the given answers in the free text fields [60]. We followed an inductive approach, e.g. the given statements from the free text fields were summarized into categories based on common themes or patterns that emerged from the data. Once categorized, the category system was discussed and revised in order to refine and validate the categories. The last step allowed for a deeper exploration of the data and ensured that the categorization accurately captured the nuances and complexities of the topic.

## Results

In total, four questions evaluating modern work environments were analysed. The number of participants, mean and standard deviation for each category are reported in Table 2. The average of each question varied slightly depending on the sector; for example, employees in the Education sector generally reported higher values compared to those in the Administrative and Support Service Activities sector. However, as there was no hypothesis about differing values depending on industries, this aspect was not analysed further.

**Table 2:** Modern work environment factors, questions, frequency (*N*), mean (*M*), and standard deviation (*SD*)

Dimension	Question	*N*	M	SD
WLB	I find it difficult to balance my professional and private life.	3308	2.28	1.11
Boundary permeability	I also work outside regular working hours, i.e. during my free time.	3235	2.25	1.02
New technologies	My workload has increased due to new technologies (email, chat, servers, cloud, ERP systems, controlling systems).	2894	2.93	1.25
WFH	I can work productively and efficiently in home office. *	1920	1.91	1.06

* This item was added later in 2020

### Work-life balance

**Fig. 1:**
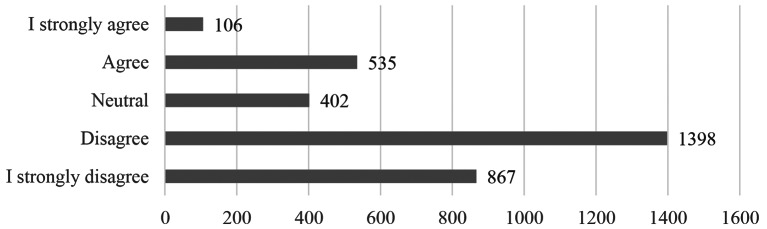
Results of the work-life balance question (*“I find it difficult to balance my professional and private life. ”*), *N* = 3308

**Fig. 2:**
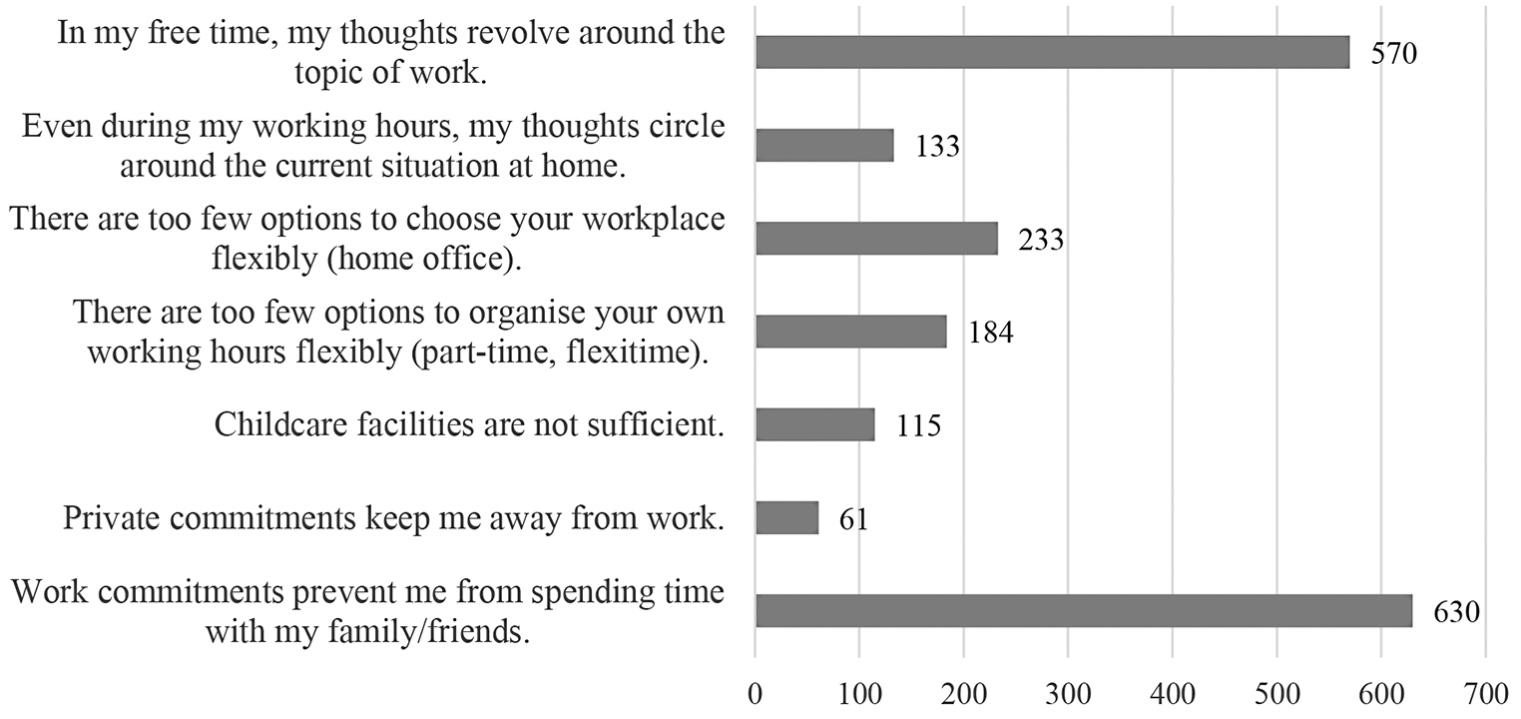
Reasons for problems with work life balance derived form the Problem question (*N* = 1043 valid responses)

Overall, many of the participants in the study were able to balance their professional and private lives (*M* = 2.28, *SD* = 1.11). The majority (68.5%) of participants had no difficulty balancing WLB, 12.2% remained neutral, and a small proportion (19.4%) reported to have difficulty, see Fig. 1 for more details. Among participants who struggled with WLB (neutral or worse), the predominant reasons were: work commitments that prevented them.

from spending time with family/friends or that their thoughts revolved around work during their free time (see Fig. 2 for all other reasons given in the Problems question). Based on the retrieved data, the spillover from work to private life occurs much more frequently than the other way around. The qualitative data from the 166 free text field responses in the Problem question highlights several aspects of why people struggle with WLB. One major theme is evolving around time management. This includes the common reasons such as employees working overtime, work at the weekends or struggle with shift work. Some participants have little time flexibility or are bound to the workplace for other reasons for example because they are cooperating across different time zones. Sometimes company etiquette is blamed, if appointments are not kept, double booked or are scheduled outside normal working hours. Participants that live far away from their workplace struggle because the long commute reduces their spare time. With regard to modern work environments, the dissolution of work boundaries and constant accessibility lead to non-compliance with the usual working hours. Several participants report spillover effects in both directions. Families struggle to balance private and professional appointments (“Due to the care of own children in daycare and primary school in combination with regular evening (work) appointments lead again and again to overlaps of professional and private appointments.”). Many participants report that they “take work home with them,” meaning that they either frequently still think about work in their free time or are still in touch with work in some way (e.g., by receiving messages afterhours). Another major theme refers to work organisation. Participants complain that many tasks and appointments are scheduled at short notice, leaving them with a heavy workload and with little time to resolve it. This often leads to overtime and, as a result, too little time to rearrange their private plans. Especially for participants with children or dependents to care for this is a difficult balancing act. Participants report an imbalance between workload and number of workers and an unsatisfactory regulation of overtime reduction. Many participants wished to have the opportunity to work remotely, and they had a desire for more flexibility: “Mobile working has to be more flexible these days. When and where must be possible at any time. Especially nowadays, it must not be dependent on the room layout at home. After all, teachers also work at home and do not have to have any premises.”. In contrast, in practice there are sometimes expectations from superiors regarding the choice of work location, e.g. several participants felt implicitly pressured by their superiors to come to the office.

### Boundary Permeability

**Fig. 3:**
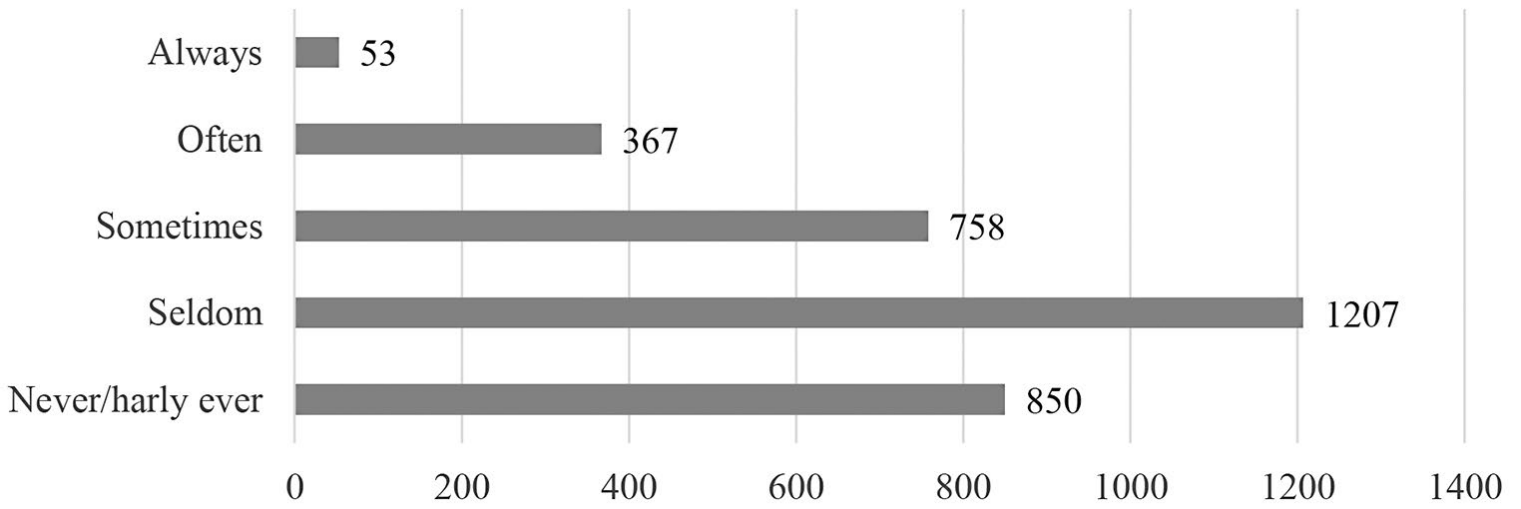
Results of the boundary permeability question (*“I also work outside regular working hours, i.e. during my free time”*), *N* = 3235

The majority (63.6%; seldom or never) of the participants did not work outside regular working hours (*M* = 2.25, *SD* = 1.02). A small proportion occasionally (23.4%) work outside regular working hours and 12.9% do this always or often (see Fig. 3).

**Fig. 4:**
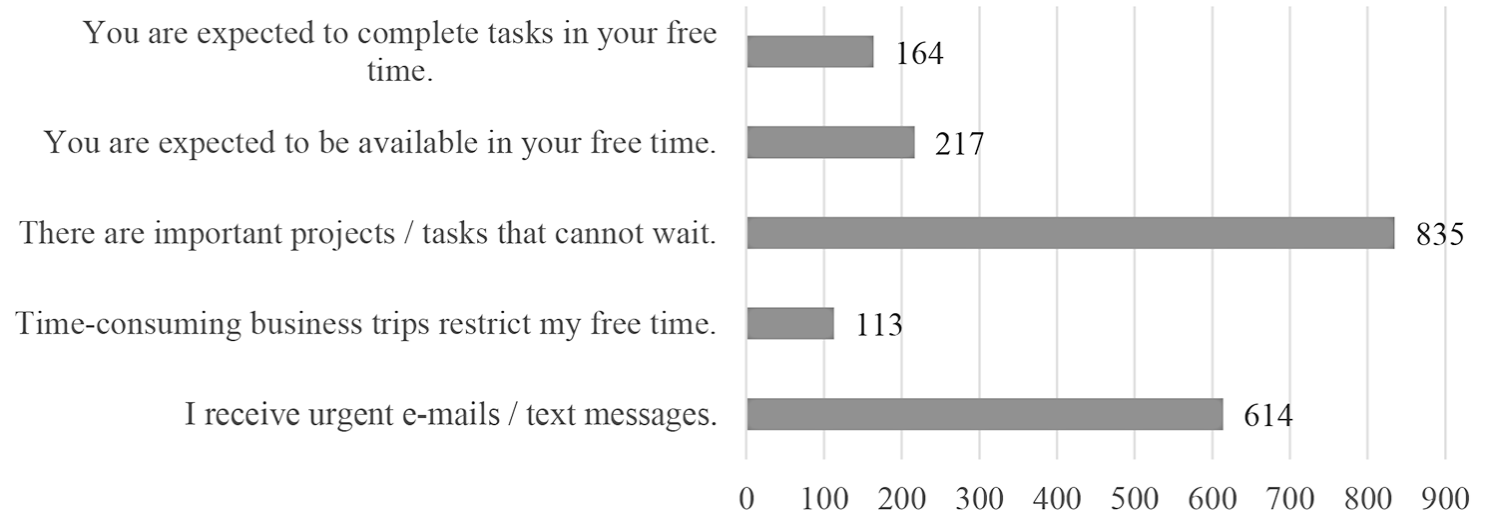
Reasons for problems with Boundary Permeability (*N* = 1178 valid responses)

The most common reason for the boundary permeability were either important tasks/projects that could not wait or the receipt of important messages (see Fig. 4). The analysis of the 210 free text field responses of participants who sometimes, often, or always struggle with work boundaries showed a few more insights. An important factor is whether the dissolution of work boundaries is voluntarily or forced without alternative. Employees struggle to find undisturbed time through their regular working hours. As a result, they revert to time outside to get work done that requires high concentration and no disturbance (“After hours, you can work more quietly and with more concentration”). As many report a heavy workload, they use time before and after work to prepare/post-work tasks. Some participants complain that calls are often scheduled outside their regular working hours. Moreover, only after hours there is enough space to think: “I think about projects and develop alternative solutions”. Some use the time outside work to enhance their professional development, for which there is otherwise no time. Time management plays an important role – on one hand, they value flexible working hours - on the other hand it becomes difficult to ever finish with work: “The boundary is fluid, i.e. it’s great that you can freely divide your time. The downside is that you always have the feeling that you’re not finished and haven’t met the requirements for working time and content.” Exceptional events with work peaks, the lack of resources and the organization of shift work are other named reasons for problems with boundary permeability.

### New technologies

**Fig. 5:**
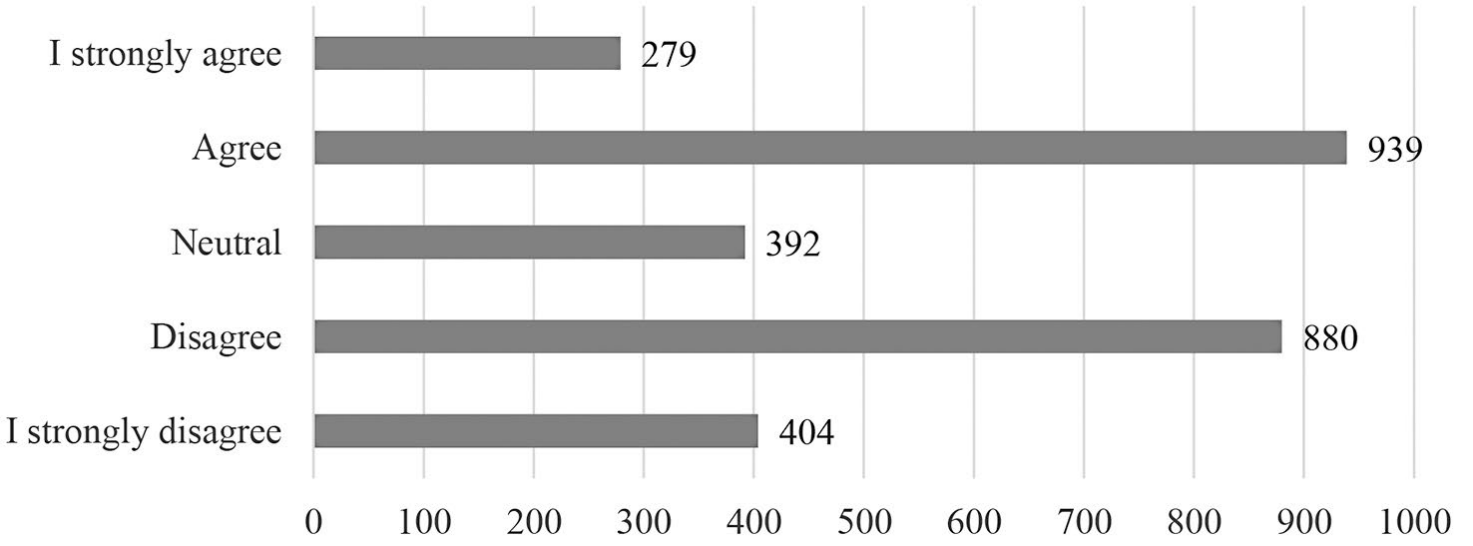
Results of the new technologies question (*“My workload has increased due to new technologies (email chat, servers cloud, ERP systems, controlling systems”*), *N* = 2894

Overall, the analysis of the new technologies question reveals that many participants see an increase in their workload due to new technologies (*M* = 2.93, *SD* = 1.25). A closer look at the data reveals two peaks: 30.4% of participants disagree with the question and 32.4% agree (see Fig. 5).

**Fig. 6:**
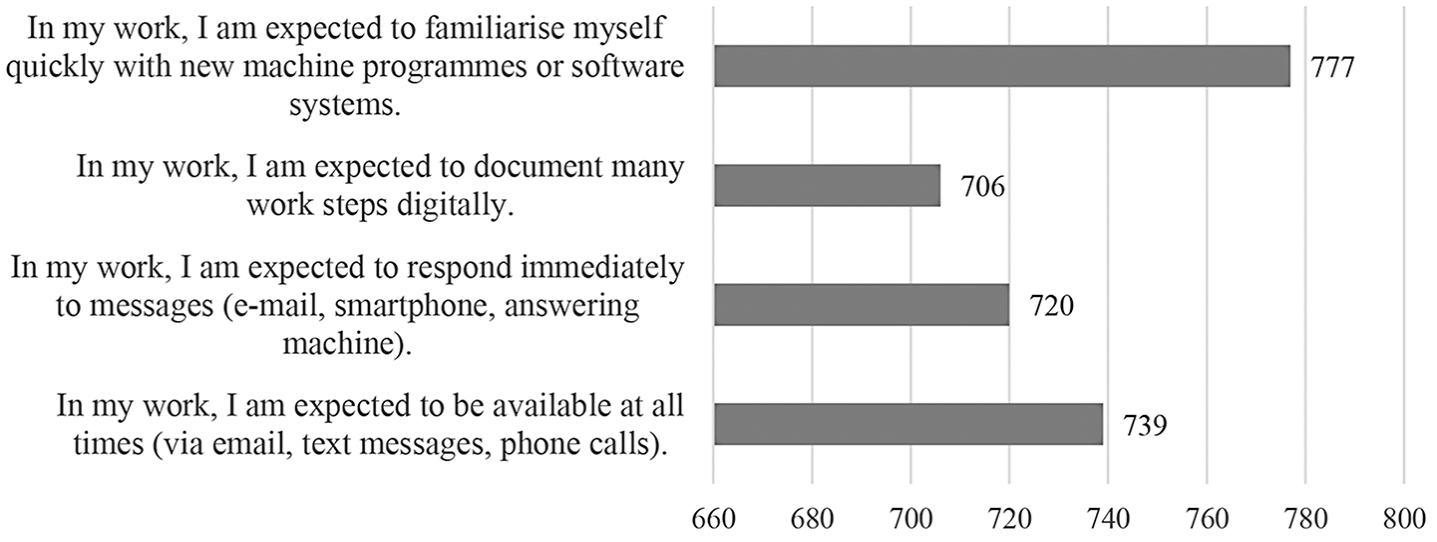
Reasons for problems with new technologies (*N* = 1610 valid responses)

High scores are justified by the fact that they are expected to quickly familiarize themselves with new programs or software systems and that they are expected to be available at all times (see Fig. 6). The evaluation of qualitative data (204 free text field responses) shows that employees see an increase in work related tasks: Too many emails, chats and programs lead to the perception of a higher workload and time expenditure. More specifically, the participants report too much chat and emails, too many programs, and, as a result an increased information input (“The work changes into new systems and old ones. Double the knowledge = double the time but not the time you get”). As a result, there is more documentation and distributed information that have to be processed. The handling of the new technologies must be learned for which there is not always time within regular working hours. Additionally, participants are constantly accessible and fast response times are expected (“you can be reached more quickly, and everyone can see if you are there [online]. As a result, many inquiries come directly via chat, and they all want an answer straight away.”). Work interruptions and technical problems lead to delays.

### Working from Home

**Fig. 7:**
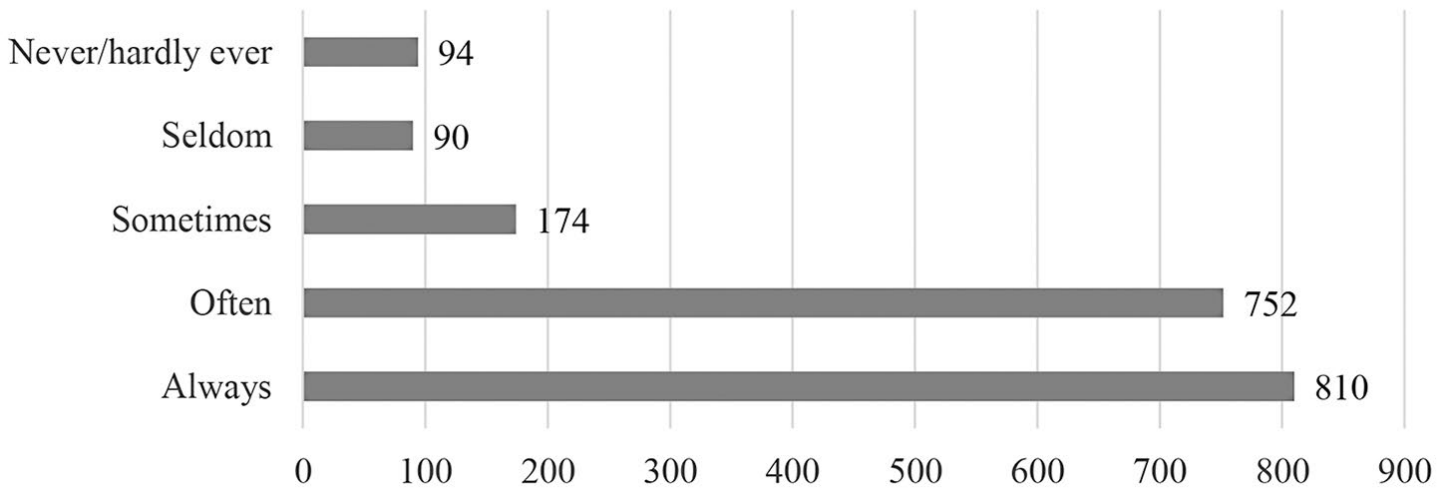
Results of the WFH question *(“I can work productively and efficiently in home office”)*, *N* = 1920

The vast majority (81.4%) of participants report that they can often or always work productively and efficiently in home offices (*M* = 1.91, *SD* = 1.06). A very small proportion (9.6%) work inefficiently from home, see Fig. 7 for more details.

**Fig. 8:**
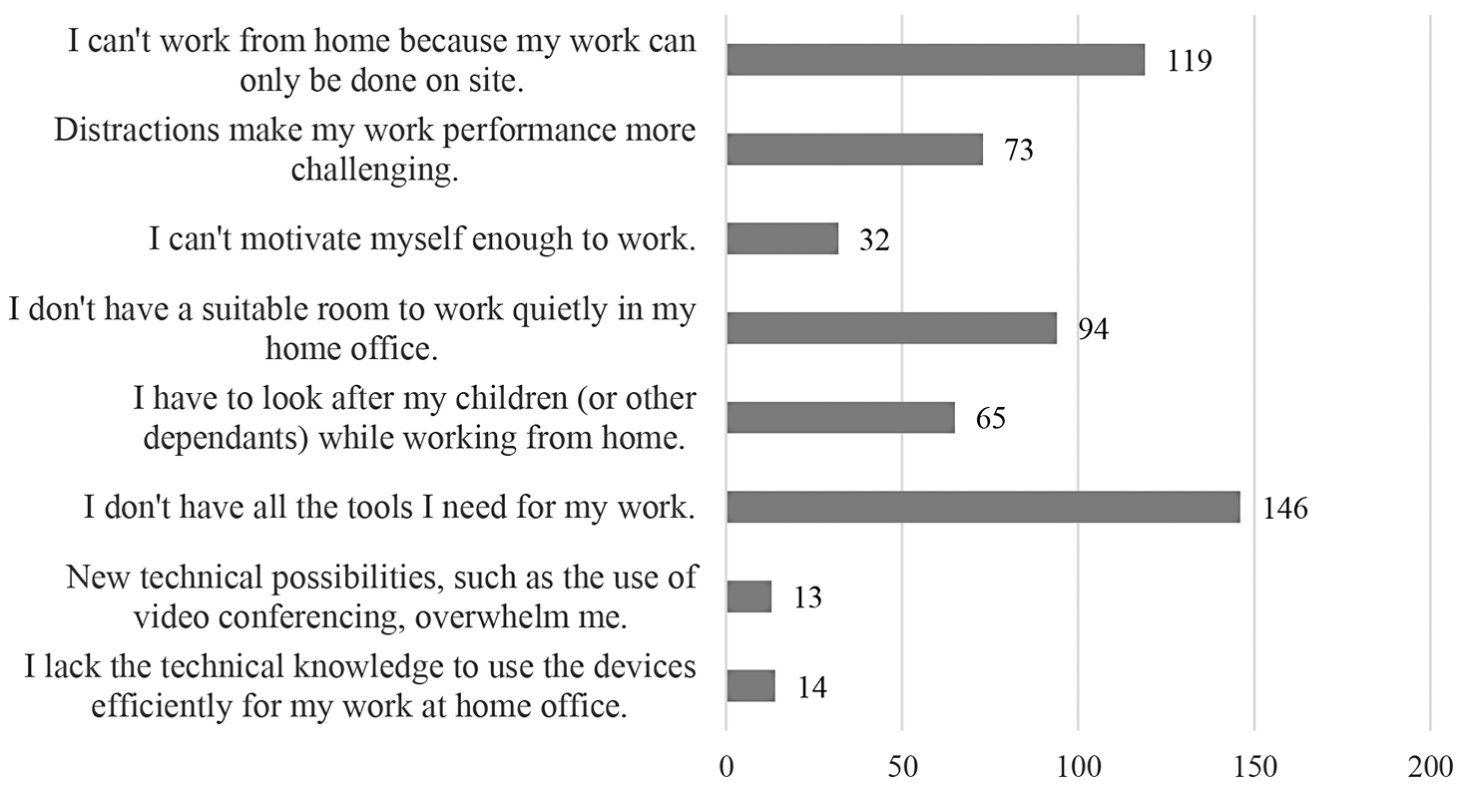
Reasons for problems with WFH (*N* = 358 valid responses)

This is due to a lack of work equipment, adequate space, or because the work cannot be done in WFH; see Fig. 8 for more details. Analysing the 86 free text responses reveal that problems with WFH occur most commonly if the technical background is not adequately met. Technical problems or insufficient digitization make it difficult to perform all work-related tasks, e.g. some participants have no access to necessary data or equipment (“Lack of access to printers and file forwarding. Company cell phones or data packages for mobile work are not provided.”). Working from home encourages the dissolution of work boundaries, which can lead to the problems described above. Many participants complained that they generally either cannot or are not allowed to work from home. Furthermore, some companies do not have uniform regulations for working from home. It appears that employees negotiate different agreements with their superiors, which seems unfair to others with different arrangements (“For me, working from home was out of the question, other colleagues simply decided this for themselves without consultation”). Some participants feel that they can work more efficiently at the office (“Face-to-face meetings are important for effective work performance and social contact”). This is partly due to the fact that the social interaction with colleagues is better there. However, this depends very much on personal preference; other participants simply preferred to work undisturbed at home.

### Solutions to improve modern work environments

Participants were asked what can be done to improve the situation from their point of view. We analysed the data of 178 free text responses with MAXQDA and summarized the answers in categories, see Table 3 for the outcome. By applying an inductive approach, a total of 6 main categories were identified: Time Management, Interaction with Technology, Behaviour of Managers, Work Organization, Stress Management, and Equal Respect for Work and Private Life. The first four categories therefore fall into the area of organisational-level solutions, while the last two concern person-related solutions. Further aspects of each category as well as exemplary verbatim quotes are reported in Table 3. A large proportion of the suggestions related to time management and time relief (114 mentions). The second most frequently mentioned category was Work Organization (46 mentions), followed by Equal Respect for Work and Private Life (14 mentions) and Behaviour of Managers (13 mentions).

**Table 3:** Category system for solutions to improve modern work environments

Main category	Subcategories	Verbatim quotes
Time Management	• Adhere to contractual working hours • Avoid unnecessary communication • Improve shift work and overtime regulations • Have a say in time allocation • Less time pressure • Improved substitution arrangements • No correspondence outside working hours • Forward and transparent planning of working hours • Offer greater choice of working time models • Find arrangement for field service • Counteracting the boundary permeability	“Away from the ideal that a good employee always works a lot of overtime and is always available.” “Many colleagues would probably like to have more flexible working hours. This should be considered in order to motivate and promote performance.”
Work Organization	• Clarify responsibilities • Appropriate amount of work tasks • Enable remote work / home office • Equitable distribution of work tasks • More or better training	“Work often goes where it is done. It would be desirable if additional tasks/project work were distributed more evenly among employees.” “Short digital training courses, instead of instructions by e-mail to teach yourself.”
Behaviour of Managers	• Uniform regulations for all • Adequate assessment of work performance • Improve leadership skills • Managers acting as role models	“Performance should be evaluated according to actual performance and not according to personal presence, then the employee can also leave at 15:00 without having a guilty conscience.”
Interaction with Technology	• Clarify needs • Improve technical equipment • Improve digitalization • Acquisition of work equipment	“In principle, it is assumed that new “systems” will simplify working life. However, the extent to which this is the case is not examined. Instead of imposing systems, one should first ascertain the current situation and then decide together.”
Equal Respect for Work and Private Life	• Consideration of private life • Less flexibility required	“Work/family balance, especially as a parent with young children and primary school children. More consideration for field work among both female and male colleagues”
Stress Management	• Strengthening personal competencies • Sports activities	“That you can switch off better and not think so much about work.”

## Discussion

This study followed several objectives. This first aim was to demonstrate that modern work environments can cause psychosocial stress, and the second objective was to describe the severity of it. We analysed the questionnaire data of 3431 workers employed in 33 different companies. On a scale from one to five, all modern work environment items were on average rated at a low to medium level (*M*-range: 1.91–2.93). This means that no evaluated aspect in this study was generally perceived as stressful by all participants but was nevertheless present for some participants. The third objective was to understand the reasons for increased stress levels better. For each factor of the modern work environment, detailed reasons are given on a quantitative level as well as additional reasons given by the participants in the free text fields. Finally, the fourth objective was to explore what can be done to reduce stress caused by modern work environments from the employees’ point of view. There were varied suggestions concerning time management, interaction with technology, behaviour of managers, work organization, stress management, as well as equal respect for work and private life.

### Modern work environments

Overall, the modern work items were rated at a medium level. Analysing WLB, it was discovered that 19.4% of the participants had difficulties. The main reasons were to be found in time management, respecting both work and private life equally, and in work organisation. 12.9% participants work always or often outside their regular working hours. Reasons for the dissolution of work boundaries were a generally high workload, undisturbed working time, and scheduled appointments afterhours. The analysis of the new technology question is interesting because the answers were two-peaked: New technologies were not a problem for around half of the sample but were for the other half. Struggling participants reported an increased information input due to new technologies, having to learn the handling of new technologies while not having the time for it, and expected fast response times with time pressure as a result. WFH was the least problematic question in this sample; the vast majority (81.4%) of participants felt they can work productively from home. When interpreting these results, one should consider that PRA was carried out in departments or other meaningful organisational structures of the individual firms. That means that the results within these units might have been high for all participants of the unit or not at all present. Taking the results of all companies together to find systematically elevated values, the mean does not show critically high values but indicates that it is problematic for part of the sample.

Although not many studies have specifically focused on modern work environments, the results of this study are reflected in the literature [61]. The mean values are comparable to other reported values, e.g. the mean value (2.28) for WLB falls within the baseline range reported by Diebig et al. [58]. Several studies report that new technologies and thus modern work environments resemble a double-edged sword: On one hand the new methods make life easier; on the other hand they facilitate boundary permeability and work intensification [37, 62–64]. For example, new communicative communication tools offer employees the flexibility necessary to manage their work and personal commitments, but equally serve as a catalyst for inducing work-life conflict [65].The forth industrial revolution is advancing and the use of new technologies is rapidly continuing. It is therefore a must for companies to face the changes and prepare their employees with support and additional knowledge how to handle new technologies [66]. With regard to the first three objectives of the study, it can be demonstrated that modern work environments can cause psychosocial stress with many underlying aspects. To detect them and assess the severity, specific questions must be asked by including them in screenings or any other method of analysis.

### Suggestions how to improve modern work environments

The last big objective of the study was to involve employees by finding solutions to manage psychosocial stress caused by modern work environments. Time management was a frequently mentioned topic. Some approaches to a solution go in the direction of stricter adherence to working hours and the separation of work and private life (“Adhere to contractual working hours”); on the other hand, some would like to see more flexible working time models (“Offer greater choice of working time models”). This may look like a contradiction on the surface, but the decisive factor is that the control or decision lies with the employee and not with the management. In general, a certain right to have a say in the allocation of working hours was a very important point in this study. This is comparable to the finding that adhering to employees’ autonomy and segmentation preferences is an important key to deal with modern work conditions [67–70]. Moreover, employees like to know with a little time lag what is coming (“Forward and transparent planning of working hours”), and spontaneous assignments or additional work are difficult to reconcile with family obligations (“Improved substitution arrangements”). These findings go in line with existing stress model such as the Job Demand Control Model [71, 72]. It states that work stress is reduced when employees have sufficient decision latitude. According to the model, especially employees with high demands and low control would benefit from interventions that allow more co-determination [73, 74]. We suggest based on our findings and existing literature that employees may benefit greatly if their self-efficacy was promoted, for instance by allowing them more autonomy and control over their work schedules [23]. This could be archived by facilitating varying schedules in company practice. For example, a 40-hour week does not necessarily have to evolve around nine to five from Monday to Friday but could be separated in varying time slots. This may include taking longer breaks during the day to exercise outdoors in daylight [75], working longer days to have long weekends off [76], or supplementing their partner’s working hours to look after the children. It can be assumed that most employees only have the confidence to do these things if they are either exemplified by their superiors or if they are repeatedly encouraged to take up these offers (see paragraph about “Behaviour of managers”). Compressed workweeks have been suggested as an alternative in the literature but were not explicitly mentioned by the employees we surveyed. The method is controversial anyway because it offers more free time in a block but can also increase the intensity of the workload. It is therefore not surprising that studies on this subject are impaired [77–79].

Employees felt that interaction with technology could be improved. They were of the opinion that some technologies are unnecessary or not used in the right context, making work more complicated rather than easier (e.g. “Clarify needs”). Many criticized the incomplete technical equipment or programs that do not work well (“Improve technical equipment”, “Improve digitalization”, “Acquisition of work equipment”). It has been demonstrated that technology use at work can cause occupational stress with effects such as a fast paced job and more interruptions and multitasking requirements [80]. It would therefore be very wise to consider if certain technologies or programs are really necessary. For the ones that are, employees would benefit greatly if they were trained in its use and the programs were designed in a user friendly way [81].

Employees attributed some importance to the behavior of managers. They detected inconsistencies, for example a lack in uniform regulations. It has been shown that the perception of equality is very important for individuals and can have a significant impact on their health if it is not given [82, 83]. Participants wished for improved leadership skills and managers as role models. The health-promoting effect of positive role models has been demonstrated [84]. Koch et al. [85] showed that employees who had role models who promoted work-life balance were more inclined to separate their work and personal lives themselves, leading to lower feelings of exhaustion and disengagement.

Further suggestions related to an improved work organization, such as clarified responsibilities, a manageable workload, and an equitable distribution of work tasks. According to the Job Demands–Resources Theory [86, 87] work pressure caused by high job demands will increase the risk of burnout and other health problems. It is therefore very important to keep an eye on the general workload and the fair distribution across all employees [88].

On the side of the person-related solutions, there were suggestions for stress management and consideration of private matters. Many employees feel they would benefit from improving their personal stress management skills. The positive effects of such programs have been demonstrated many times over [89–91]. In addition, sporting activities would be a welcome addition to health measures [92, 93]. As many employees for instance work in a seated position, they lack regular physical activity. The opportunity to exercise at work would make it much easier for them to integrate it into their lives. Considering private matters would mean that employers are respecting regular working hours (no late calls, etc.) and do not demand to be available outside regular working hours. The negative impact of too much interference of work with private life has been shown; a high work-home interference, for example, can lead to depressive symptoms [94]. It would therefore be advantageous if companies made it easier for their employees to maintain the boundary between work and private life.

Many proposed solutions in this study have in common that they focus on the result, not on a measure itself. For example: “That you can switch off better and not think so much about work” is the result that the employee wants, but it is still completely open as to how it can be achieved. To get more concrete solutions it would still be advisable to hold workshops with the employees and to tailor the measures to their specific setting. In the vast field of possible measures and interventions it is almost impossible to find general, easy to apply solutions that would fit to most company settings. In summary, one promising solution to dealing with the potential negative effects of modern work environments would be for companies to offer a range of options while allowing employees to choose the options that suit them best. It has been demonstrated that individual work-life balance strategies can work well [95]. Furthermore, personal segmentation preferences are crucial when it comes to managing WLB – managing one’s own boundaries has shown promising results in improving WLB [37, 96, 97]. This means, for example, offering flexible working arrangements but still finding a way to meet overall contracted working hours [98]. For instance, it could be agreed that there are fixed times or days on which employees must be in the office/available, and the rest of the time employees can organize their working hours as they wish. Digital tools offer great opportunities to realize this endeavour, for example by sharing calendars or using time accounts.

### Practical implications

This study offers many aspects and details to better understand the complex field of modern work environments. Many possible solutions for dealing with the negative impact were described and discussed. When designing organisational interventions, PRA and other assessments, one can obtain many ideas and starting points to be gained from this study, in order to ask the right questions and to develop solutions that resonate with employees. As every company, every department and every field of expertise is different, it is nevertheless essential to consider the specific context and involve the employees concerned.

### Limitations

The findings of this study should be understood in light of potential limitations. Firstly, the participation was voluntarily and therefore a selection bias cannot be ruled out. Due to varying recruitment strategies and internal company recruitment methods, we lack information on the number of participants invited, making it impossible to calculate a response rate. We recorded one measurement point in time, so we cannot depict a time course. However, as the data collection covers a period of more than four years, this study is not subject to the typical limitations of a cross-sectional study in this regard. The qualitative approach allows a detailed insight and enables the exploration of new approaches. However, qualitative data is subject to limitations stemming from subjectivity on both ends: the participants and the interpreter. All results are based on self-reported data in an online questionnaire. It is therefore possible that responses were influenced by factors such as the design of the questionnaire or social desirability [99]. This study presents the employees’ perspective on modern work environments – it would be a valuable extension to consider the employers’ perspective as well.

## Conclusion

The impact of modern work environments is a serious issue in its own right within the psychosocial risks in the world of work. There are several aspects, such as WLB, boundary permeability, new technologies and remote work, that should be considered. We argue that modern work environments should be regularly assessed in PRA. Offering solutions would not only prevent risks to employees’ health, but also put the company on a good path for future challenges. As change is in the nature of modern work environments, it is particularly important to regularly review employees’ stress levels and counterbalance them where necessary. This not only requires companies to carry out regular screenings, but also policymakers and healthcare experts to adapt PRA accordingly and offer suitable solutions. To date, there are not enough scientifically sound measures to counteract the negative effects of modern work environments.

## Data Availability

The category system is available from the corresponding author on reasonable request.

## Abbreviations

PRA: Psychosocial Risk Assessments
WFH: Working from Home
WLB: Work Life Balance
